# The synergistic anticancer effect of formosanin C and polyphyllin VII based on caspase‐mediated cleavage of Beclin1 inhibiting autophagy and promoting apoptosis

**DOI:** 10.1111/cpr.12520

**Published:** 2018-10-18

**Authors:** Jingxia Cui, Shuli Man, Nina Cui, Li Yang, Qianbei Guo, Long Ma, Wenyuan Gao

**Affiliations:** ^1^ Key Laboratory of Industrial Microbiology, Ministry of Education, Tianjin Key Laboratory of Industry Microbiology, State Key Laboratory of Food Nutrition and Safety, National and Local United Engineering Lab of Metabolic Control Fermentation Technology, College of Biotechnology Tianjin University of Science & Technology Tianjin China; ^2^ Tianjin Key Laboratory for Modern Drug Delivery and High Efficiency, School of Pharmaceutical Science and Technology Tianjin University Tianjin China

## Abstract

**Objectives:**

Drug combination has a promising and potential development prospect in the treatment of various cancers. The objective of this study is to investigate the synergistic mechanisms of polyphyllin VII (PVII) and formosanin C (FC) in lung cancer.

**Materials and methods:**

The combination of FC and PVII influenced on the apoptosis, autophagy, and the relative signalling pathways were analysed in lung cancer cells.

**Results:**

The combination of FC and PVII demonstrated a concentration‐ dependent growth inhibition in human lung cancer cells. The combination index (CI) obtained from four lung cancer cells was smaller than 1. This synergistic antitumour effect was based on the increase of their single proapoptotic effect but inhibiting FC‐induced autophagy in NCI‐H460 cells. FC and PVII activated proapoptotic elements like cleaved‐caspase‐3, ‐8, and ‐9 to induce Beclin1 cleaved into Beclin1‐C which suppressed FC‐triggered autophagy and enhanced apoptosis.

**Conclusions:**

Formosanin C and PVII showed a synergistic antitumour effect on lung cancer cells. The findings would provide the foundation for the use of combination drugs in the future.

**Abbreviations**
3‐MA: 3‐methyladenine
AO: acridine orange
AVOs: autophagic acidic vesicles
CI: combination indexes
CQ: chloroquine
FC: formosanin C
MTT: 3‐(4,5‐dimethyl‐thiagol‐2yl)‐2,5‐diplenyltertrazollium
PI3K: phosphatidyl inositol phosphate kinase 3
PVII: polyphyllin VII
RPS: *Rhizoma Paridis* saponins
RT: room temperature

## 1 INTRODUCTION

Lung cancer is one of the most common incident cancers and the leading cause of cancer death in the world.^1^ Currently, people pay more and more attention to the natural products based on their various compounds and multitargets in their antitumour treatment.^2^, ^3^, ^4^ In our previous research, *Rhizoma Paridis* Saponins (RPS), which was isolated from the rhizoma of *Paris polyphylla* var. *yunnanensis*, inhibited lung cancer in multimodels such as diethylnitrosamine‐induced lung cancer mice,^5^ urethane‐induced lung carcinogenesis mice,^6^ Lewis pulmonary adenoma mice^7^ and so forth.

Modern experiments discovered that RPS possessed two main structures of saponins including diosgenin and pennogenin.^8^ Polyphyllin I^9^ and formosanin C (FC)^10^ belonging to the diosgenins, exhibited the anticancer activity mainly through inducing cellular apoptosis and activating autophagy. FC inhibited Bcl‐2 and activated proapoptotic elements including Bax, caspase‐2,^11^3, and ‐9.^12^ Meanwhile, FC provided a potential inhibitive effect on pulmonary metastasis via repression of matrix metalloproteinases.^13^ Polyphyllin VII (PVII) as a pennogenin, showed anticancer activity mainly through triggering G2/M cell cycle arrest and apoptosis based on a caspase‐3‐dependent manner^14^ and inducing mitochondria dysfunction.^15^

With the wide application of complex mixtures in clinics, synergistic interactions play important roles in phytomedicine. In our previous study, RPS as a mixture, displayed a better antitumour effect than its monomers did. Two diosgenins, polyphyllin I and FC from RPS displayed a synergistic antitumour effect on hepatocarcinoma cells through increasing their single G1 phase arrest and mitochondria‐dependent apoptotic pathway.^16^ Recently, after further screening different kinds of saponins from RPS in the synergistic antitumour effect on lung cancer, FC and PVII exhibited different antitumour mechanisms and had a stronger synergistic antitumour activity in lung cancer cells. Therefore, it is of inherent importance to carry out their synergistic antitumour research.

## 2 MATERIALS AND METHODS

### 2.1 Reagents

Formosanin C and PVII were purchased from National Institute for the Control of Pharmaceutical and Biological Products, China. Their batches were 111591‐201604 and 111593‐201604, respectively. The other reagents were commercially available and of analytical purity.

### 2.2 Cell culture

Human lung cancer cell lines (NCI‐H1299, NCI‐H446, NCI‐H460 and NCI‐H520) were obtained from Shanghai Institutes for Biological Sciences, Chinese Academy of Sciences (Shanghai, China). The cells were maintained in RPMI 1640 supplemented with 10% heat‐inactivated (56°C, 30 minutes) foetal calf serum, and 1% penicillin‐streptomycin (Solarbio Science & Technology Co., Beijing, China) at 37°C in a humidified atmosphere (5% of CO_2_).

### 2.3 MTT assay

Cell viability was determined by a colorimetric assay using 3‐(4,5‐dimethyl‐ thiagol‐ 2yl)‐2,5‐diplenyltertrazollium (MTT). Four kinds of cancer cells were seeded at a density of 1 × 10^4^/well in a complete growth medium in 96‐well plates. The cells were incubated with the test compounds for 24 hours before the MTT assay. Then, a fresh solution of MTT (0.5 mg/mL) was added to each single well with a further incubation for 4 hours. Finally, the cells were dissolved with 100 μL of DMSO and then analysed in a multiwall plate reader at 570 nm (BioTek Instruments, Inc., Winooski, VT, USA).

### 2.4 Colony formation assay

The NCI‐H460 cells were digested with 0.25% trypsin and split into individual cells. Subsequently, 1000 cells were seeded into 3‐mL culture dishes and maintained under standard culture conditions for 1 week. When the colonies were visible to the naked eye, the cells were incubated with the test compounds for 5 days. Then, the colonies were stained with MTT (0.5 mg/mL) for 4 hours and observed under a microscope.

### 2.5 Median effect analysis

For combination studies, MTT assays of FC and PVII at a fixed concentration ratio (1:1) were carried out for 24 hours treatment. The concentration‐response curves of each agent for each cell line were obtained. The interactions between drugs were evaluated by calculating Chou‐Talalay combination indices (CI) using Compusyn software.

### 2.6 Annexin‐V/PI double‐staining

An FITC annexin‐V Apoptosis Detection Kit I (KeyGEN BioTECH, Nanjing, China) was used to detect apoptosis in human lung cancer cells after exposure to the test compounds. NCI‐H460 cells were washed in phosphate‐buffered saline thrice and resuspended in 100 μL of binding buffer with FITC annexin‐V and PI (1 μL each). The cell suspension was incubated for 15 minutes at room temperature (RT) in the dark, and analysed by flow cytometry (BD Accuri C6; Becton Dickinson, Franklin Lakes, NJ, USA) within 1 hour. Quantification of apoptotic cells was determined using Annexin V‐FITC and PI was used to distinguish necrotic cells.

### 2.7 Hoechst 33258 staining

NCI‐H460 cells were treated with 1 μmol/L of FC, 1 μmol/L of PVII, or a combination of FC and PVII (1 μmol/L:1 μmol/L) for 24 hours. The cells were washed in phosphate‐buffered saline thrice and then fixed in 100% ice‐cold methyl alcohol for 10 minutes. After treatment, the cells were washed in PBS thrice and stained with Hoechst 33258 (1 μg/mL) for 30 minutes. Cells were photographed by fluorescence microscope.

### 2.8 Fluorescence imaging of cells

For AO staining, NCI‐H460 cells (1 × 10^5^/well) were seeded and cultured at 37°C for 24 hours. After treatment with the test compounds of 24 hours, 2 μmol/L AO was added to the cells and incubated at 37°C for 15 minutes in the dark, the cells were washed three times with Dulbecco's phosphate‐buffered saline. Images were captured and analysed using a fluorescence microscopy (Olympus, Tokyo, Japan).

### 2.9 Immunofluorescence staining

Cells grown on glass coverslips were treated with the test compounds for 24 hours. Next, slides were fixed with 4% paraformaldehyde for 20 minutes and treated with 0.05% Triton X‐100 for 15 minutes at RT. Thereafter, the coverslips were stained with LC3 antibody (Santa Cruz Biotechnology Inc., Santa Cruz, CA, USA) (diluted in 5% BSA) for 3 hours at RT, then washed with PBS thrice, followed by incubation with secondary antibodies at RT for 1 hour. Next, nuclei were stained with DAPI for 1 minute. Images were acquired using fluorescence microscopy.

### 2.10 siRNA transfection

NCI‐H460 cells were plated into six‐well plates with 20%‐30% confluency for 24 hours. Then, the cells were transfected with 100 nmol/L siRNA using Lipofectamine 2000 (Invitrogen, Carlsbad, CA, USA). The siRNA targeting Beclin1 and the scramble control (NC) siRNA were designed, modified, and synthesized by Invitrogen.

### 2.11 Western blot assay

Cells treated with test compounds were prepared with lysis buffer containing protease/phosphatase inhibitor cocktail purchased from Sigma (St. Louis, MO, USA). The cells were lysed in 4× Laemmli sample buffer (Sigma Chemical), heated for 5 minutes at 100°C. Proteins were separated by 12% SDS‐PAGE using the Mini‐Protean Bio‐Rad II System and transferred to PVDF membrane. These membranes were blocked for 1 hour at RT with 5% skim milk powder and probed with primary antibodies at 4°C overnight on a shaker. The primary antibodies were MAP LC3α/β (G‐4), Bax, Bcl‐2 (Santa Cruz Biotechnology Inc.), caspase‐9, cleaved‐caspase‐3, cleaved‐caspase‐8 and Beclin‐1 (Cell Signalling Technology, Boston, MA, USA). After washing, blots were incubated with secondary anti‐rabbit antibody at 1:5000 dilutions for 1 hour at RT. After washing, protein bands were visualized by Odyssey infrared imaging system (LI‐COR Biotechnology, Lincoln, NE, USA). Western blot analyses were performed using standard procedures. All the experiments were repeated thrice.

### 2.12 Statistical analysis

Statistical evaluation was conducted by using SPSS 17.0 for Windows package software. Data have been expressed as the means ± standard error mean (SEM). One‐way variance analysis and Duncan multiple range test were used to determine significantly different groups. *P* values less than 0.05 were considered as significant differences for all statistical calculations.

## 3 RESULT

### 3.1 FC and PVII exerted synergistic antiproliferative effects on lung cancer cells

**Figure 1:**
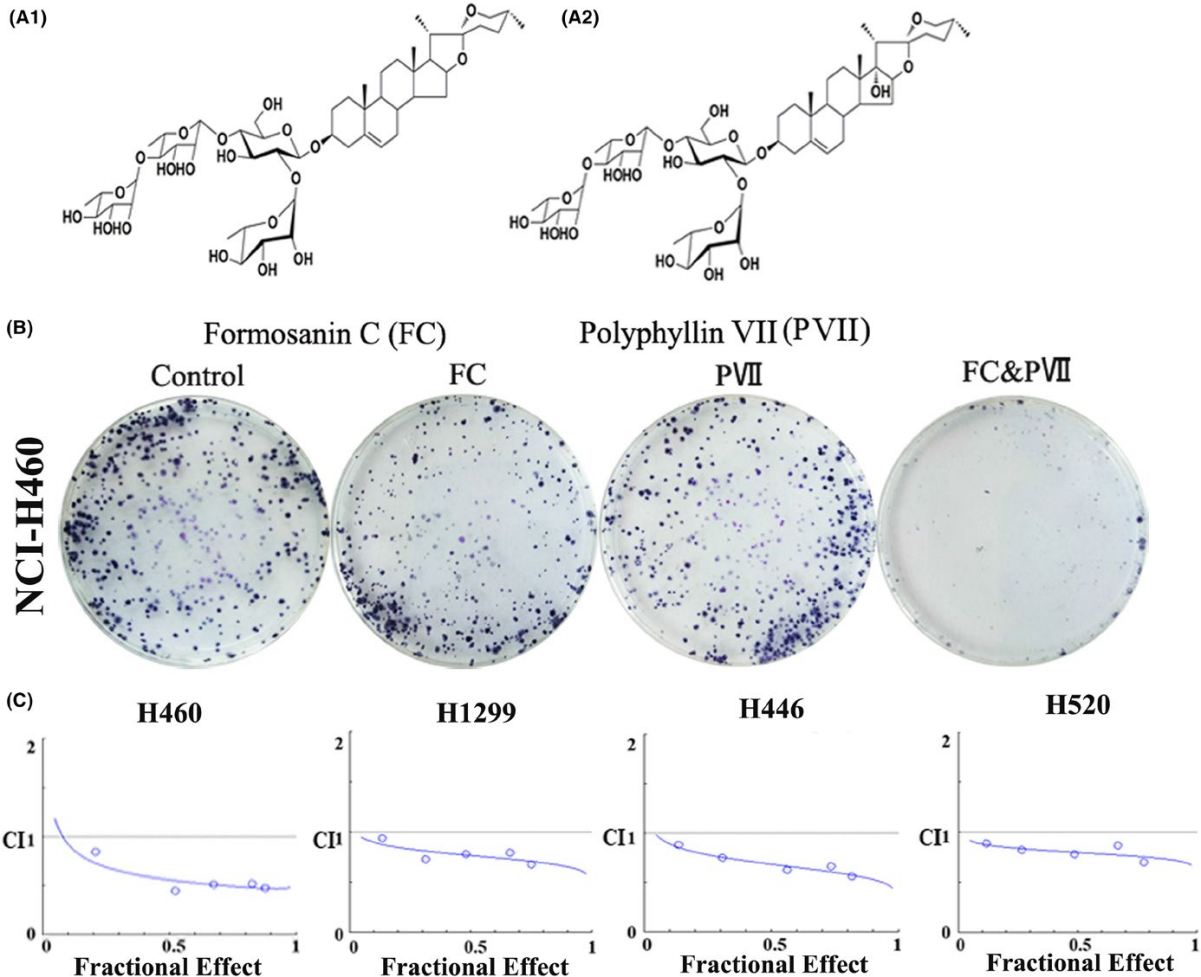
The synergistic effect of FC and PVII on different lung cancer cell lines. (A) Structures of two steroidal saponins used in this study, including (A1) formosanin C (FC) and (A2) polyphyllin VII (PVII). (B) Colony formation assay for the combination of FC and PVII. Cells were treated with DMSO, FC (1 μmol/L), PVII (1 μmol/L), and FC & PVII (1 μmol/L:1 μmol/L), respectively. (C) Growth inhibitory effects for combination of FC and PVII using the Chou‐Talalay method. The CI theorem of Chou‐Talalay defines synergism, 0.3 < CI<0.9; additively, 0.90 ≤ CI≤1.1; antagonism, 1.1 ≤ CI≤10 in drug combination. *X*‐axis, the ratio of the fraction effect; *Y*‐axis, CI, the combination index

FC and PVII (Figure 1A) significantly inhibited cell viability in four lung cancer cell lines in a concentration‐dependent manner. In order to share an equipotent effect of each partner, the combination ratios were designed to approximate the IC50 ratios. Colony formation assay indicated that there were the least cells in the combination group among four groups (Figure 1B). Subsequently, different cells were exposed to various concentrations (0.25, 0.5, 1.0, 2.0, and 2.5 μmol/L) of FC, PVII or their combination (1:1 concentration) (Table 1). The combination of FC and PVII showed a higher inhibitive rate than single‐agent treatment in each cell (Table 2). Therefore, the combination induced synergistic effects in lung cancer cells, following evaluation of the CI through isobiologram analyses. The results showed that the CI values in four kinds of lung cancer cell lines were less than 1, indicating synergistic effects between two compounds. Furthermore, the synergistic effect was the most obvious in NCI‐H460 cells (0.3 < CI<0.9) (Figure 1C).

**Table 1:** The CI values of FC and PVII in lung cancer cell lines

Concentration (μmol/L)		NCI‐H460		NCI‐H446		NCI‐H1299		NCI‐H520	
FC	PVII	Effect	CI	Effect	CI	Effect	CI	Effect	CI
0.25	0.25	0.21	0.84	0.14	0.88	0.14	0.94	0.12	0.89
0.5	0.5	0.53	0.44	0.31	0.74	0.32	0.72	0.27	0.82
1	1	0.68	0.50	0.56	0.62	0.48	0.77	0.49	0.78
2	2	0.83	0.52	0.74	0.66	0.67	0.79	0.67	0.86
2.5	2.5	0.88	0.47	0.82	0.55	0.75	0.67	0.78	0.88

**Table 2:** The IC50 of FC and PVII in lung cancer cell lines (μmol/L)

IC50	H460	H1299	H446	H520
FC& PVII	1.12 ± 0.11	1.77 ± 0.23	2.08 ± 0.24	2.12 ± 0.36
FC	2.13 ± 0.27**	2.87 ± 0.16**	2.73 ± 0.21*	2.87 ± 0.08*
PVII	1.99 ± 0.13**	2.59 ± 0.30*	2.46 ± 0.26	2.53 ± 0.19

*P < 0.05, **P < 0.01, significant differences compared with FC& PVII group.

The combinative concentration ratio of FC & PVII was 1:1.

### 3.2 Combination of FC and PVII enhanced their single proapoptosis in NCI‐H460 cells

**Figure 2:**
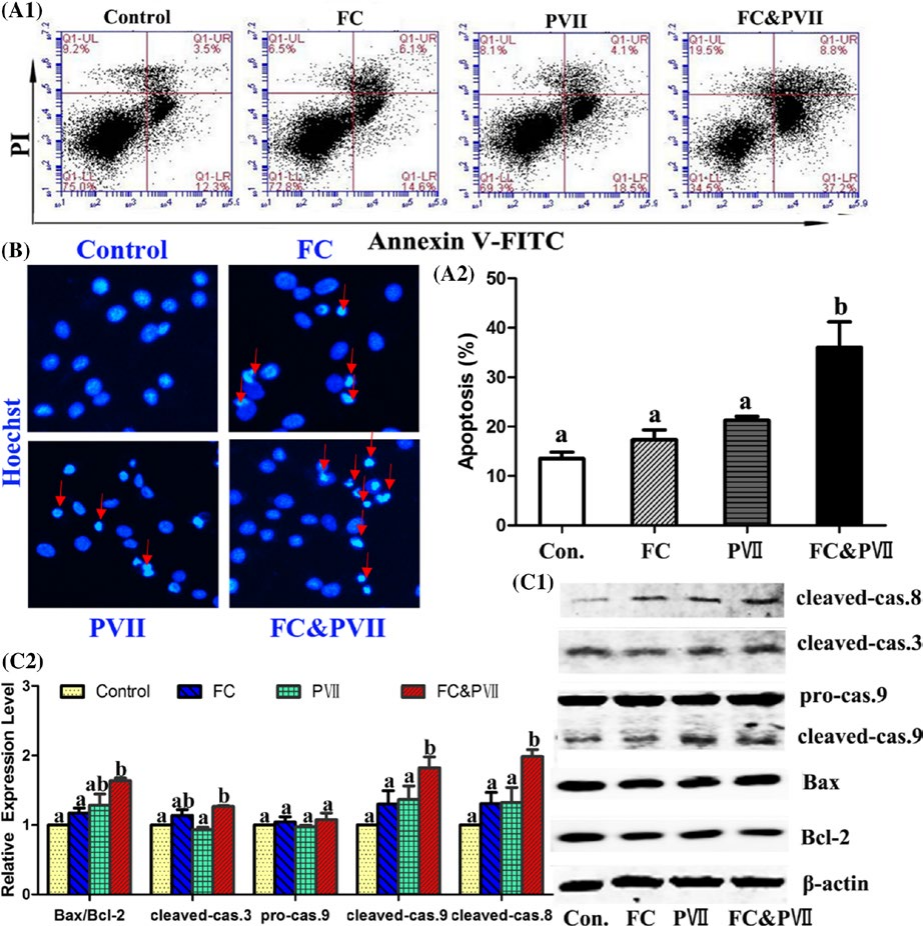
Combination of FC and PVII enhanced the induction of apoptosis in NCI‐H460 cells. Cells were treated with DMSO, FC (1 μmol/L), PVII (1 μmol/L), and FC & PVII (1 μmol/L:1 μmol/L), respectively. (A1) Annexin V/PI assay showed the apoptotic effect of FC&PVII in H460 cells. (A2) Statistical analysis of Annexin V/PI assays for apoptotic effect. The value of the *Y*‐axis was the total number of the early apoptotic and late apoptotic cells. Different letters meant significant differences between two groups (*P* < 0.05). (B) The Hoechst 33258 expression was observed by fluorescence assay. Arrows indicated that chromatin condensation and nucleus fragmentation mainly occurred in FC & PVII‐treated cells. (C1) Western blot analysis of different apoptosis regulatory proteins (Bax, Bcl‐2, and cleaved‐caspase 3, 8, 9) to confirm the induction of cellular apoptosis. (C2) Statistical analysis of western blot. Different letters meant significant differences between two groups (*P* < 0.05)

To investigate whether the growth inhibition of combination was caused by cell apoptosis, Annexin V‐FITC/PI double‐staining and Hoechst 33258 staining were employed in H460 cells (Figure 2A‐B). As a result, apoptosis rate induced by FC and PVII treatment alone showed no difference when compared with control group, while it significantly increased in the combination group in H460 cells. Meanwhile, combination drug induced more chromatin condensation and nucleus fragmentation than compound used alone (Figure 2B). Caspase cascade promoted the essential basis of apoptosis. To investigate the synergistic proapoptotic mechanisms of the combination group, the levels of pro‐ and antiapoptotic proteins were measured by western blot. Our results revealed that cotreatment with FC and PVII synergistically downregulated the antiapoptotic protein like Bcl‐2 and upregulated proapoptotic proteins such as cleaved‐caspase‐3, 8, 9, and Bax in H460 cells (Figure 2C).

### 3.3 Combination of FC and PVII inhibited autophagy induced by FC in NCI‐H460 cells

**Figure 3:**
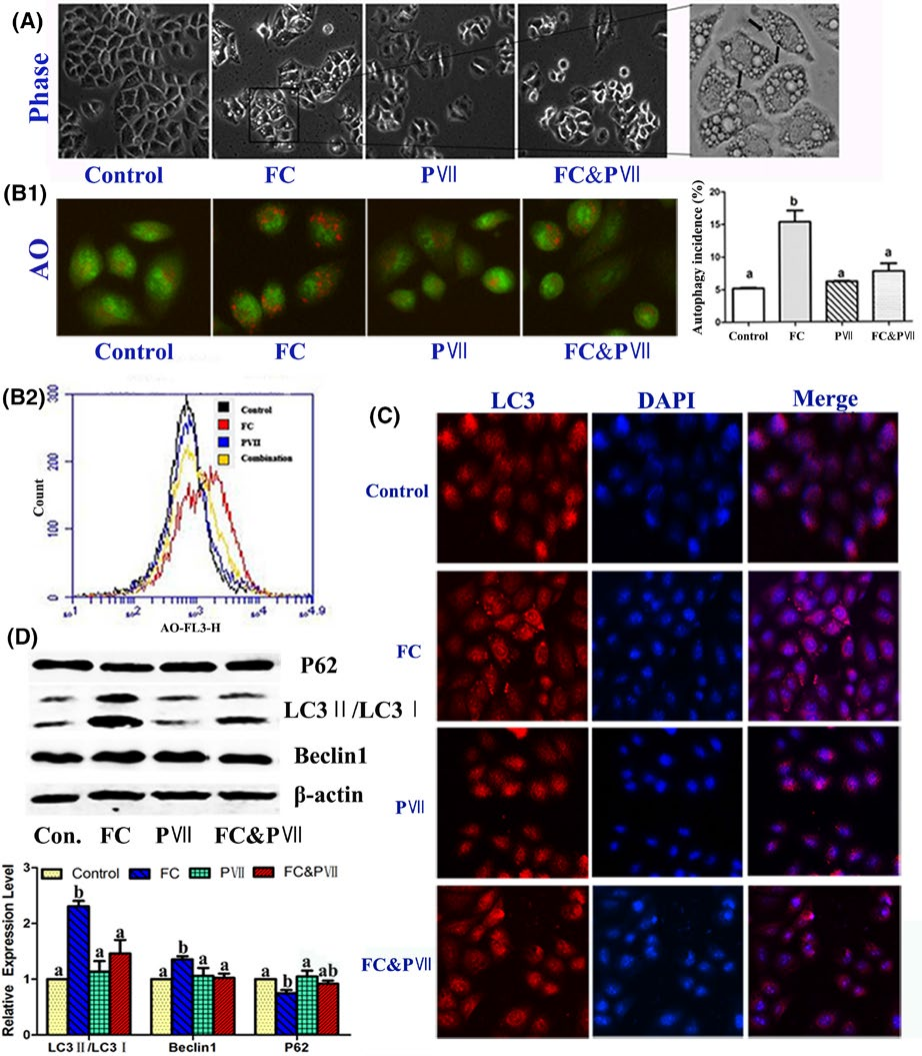
Combination of FC and PVII inhibited autophagy induced by FC in H460 cells. (A) Cells were treated with DMSO, FC (1 μmol/L), PVII (1 μmol/L), and FC & PVII (1 μmol/L:1 μmol/L) for 24 h and then observed under microscope to reveal the formation of vacuoles in cell cytoplasm. (B) Fluorescence images (B1) and flow cytometry (B2) profiles of NCI‐H460 cells treated with different agents after AO staining. The red fluorescence indicated AVOs formation stained with AO. (C) The LC3/DAPI expression was observed by immunofluorescence assay (×1000, final magnification). (D) Western blot analysis of autophagic marker proteins P62, LC3, and Beclin1. Different letters meant significant differences between two groups (*P* < 0.05)

According to previous studies, FC induced autophagy in H460 cells.^10^ To understand the mechanism of combination, cellular morphology was observed using microscope. After 24‐hours treatment with FC, numerous cytoplasmic vacuoles were formed. However, the number was decreased in the combination group (Figure 3A). Acridine orange (AO) staining was used to identify autophagic acidic vesicles (AVOs) formation. Flow cytometer quantified the red fluorescence intensity of AVOs stained with AO. As a result, the combination group decreased the number and the fluorescence intensity of the autophagic acidic vesicles induced by FC (Figure 3B). In addition, the formation of LC3 puncta was analysed by immunofluorescence staining. As shown in Figure 3C, cytoplasmic LC3 formation was observed in FC‐treated cells and disappeared in combination‐treated cells. To further make clear whether combination of FC and PVII inhibited autophagy induced by FC, the protein expression of P62, LC3‐II /I, and Beclin‐1 which were regarded as the autophagy makers were determined by western blotting. As a result, protein expression of LC3‐II and Beclin‐1 was increased by FC and sent back nearly to the normal by the combination group (Figure 3D). P62 displayed the opposite trend. These results suggested that FC induced autophagy and combination of FC and PVII inhibited autophagy in H460 cells.

### 3.4 The role of PVII played in the inhibition of autophagy was not the same as that of CQ or 3‐MA

**Figure 4:**
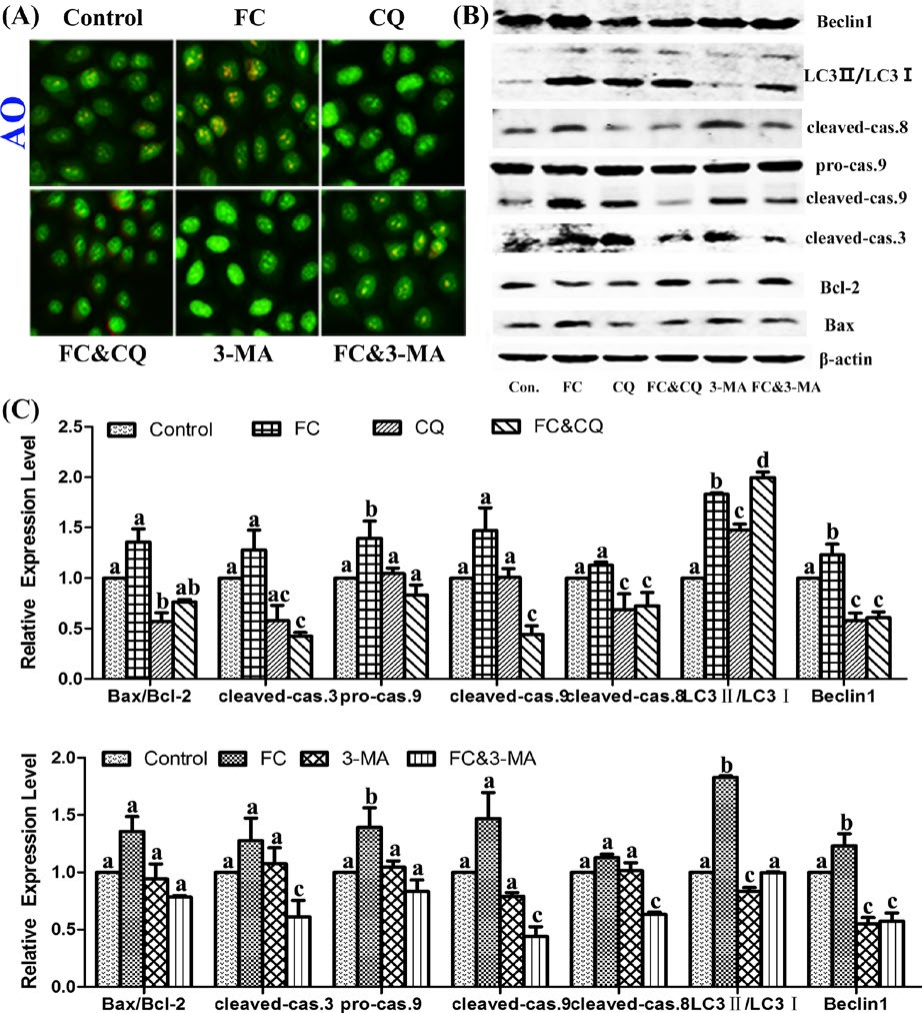
Inhibitors of autophagy suppressed apoptosis induced by FC in NCI‐H460 cells. Cells were treated with DMSO, FC (1 μmol/L), CQ (10 μmol/L), 3‐MA (10 mmol/L), FC & CQ (1 μmol/L:10 μmol/L), FC & 3‐MA (1 μmol/L:10 mmol/L) for 24 h. (A) Fluorescence microscopic images of AVOs formation were stained with AO, which appeared red fluorescence. (B) Western blot analysis of different apoptosis regulatory proteins (cleaved‐caspase 3, 8, 9, Bcl‐2, and Bax) and autophagy proteins (LC3 and Beclin1). (C) Statistical analysis. Different letters meant significant differences between two groups (*P* < 0.05)

To understand the antiautophagy role of PVII playing in the combination of FC and PVII, 3‐methyladenine (3‐MA) as the inhibitor of autophagy initiation protein class III phosphatidyl inositol phosphate kinase 3 (PI3K) and chloroquine (CQ) as the inhibitor of later period autophagy involved in the fusion of autophagosome and lysosome were used as the control to compare their reactions with FC. The results manifested that 3‐MA and CQ significantly decreased apoptosis protein expression of cleaved‐caspase‐3, ‐8, and ‐9. The autophagic inhibitors could not enhance the apoptosis induced by FC. The variation trend of the apoptotic proteins was not the same as that induced by PVII & FC (Figure 4).

### 3.5 Caspases inhibitor potentiated the effects of FC or FC&PVII on the induction of autophagy and inhibition of apoptosis in NCI‐H460 cells

**Figure 5:**
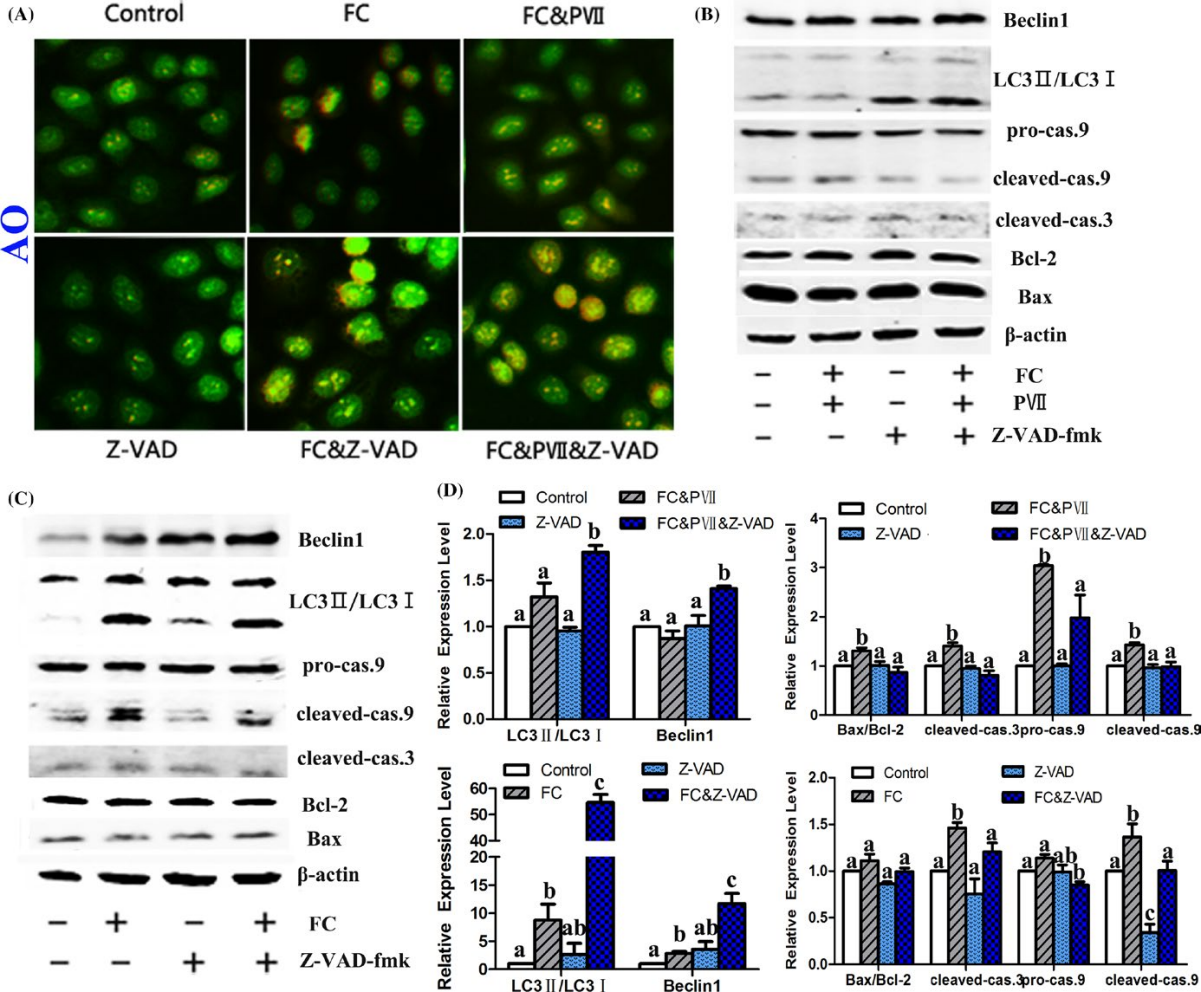
Combination of the pan‐caspase inhibitor Z‐VAD‐fmk with FC or FC&PVII promoted autophagy in NCI‐H460 cells. Cells were treated with DMSO, FC (1 μmol/L), FC & PVII (1 μmol/L:1 μmol/L), Z‐VAD‐fmk (40 μmol/L), FC& Z‐VAD‐fmk (1 μmol/L:40 μmol/L), and FC & PVII & Z‐VAD‐fmk (1 μmol/L:1 μmol/L:40 μmol/L) for 24 h. (A) Fluorescence microscopic images of AVOs formation were stained with AO, which appeared red fluorescence. (B, C) Western blot analysis of different apoptosis regulatory proteins (cleaved‐caspase 3, 9, Bcl‐2, and Bax) and autophagy proteins (LC3 and Beclin1) in FC&PVII (B) or FC (C) combining with Z‐VAD‐fmk groups. (D) Statistical analysis. Different letters meant significant differences between two groups (*P* < 0.05)

To investigate the proapoptotic role of PVII in the synergistic effect of FC&PVII in NCI‐H460 cells, a pan‐caspase inhibitor Z‐VAD‐fmk was further applied. The results manifested that Z‐VAD‐fmk significantly decreased the protein levels of cleaved‐caspase‐3 and ‐9. However, it promoted the formation of autophagic vacuoles (Figure 5A) and increased the autophagy protein levels of LC3II/LC3I and Beclin1 (Figure 5B,C). These results manifested that the balance between apoptosis and autophagy was associated with caspase regulation. Furthermore, combination of FC and Z‐VAD‐fmk‐treated cells displayed the similar phenomenon to that in the combination of FC&PVII and Z‐VAD‐fmk groups.

### 3.6 Combination of PVII & FC induced caspases mediating cleavage of Beclin1, which terminated autophagy and promoted apoptosis induced by FC

**Figure 6:**
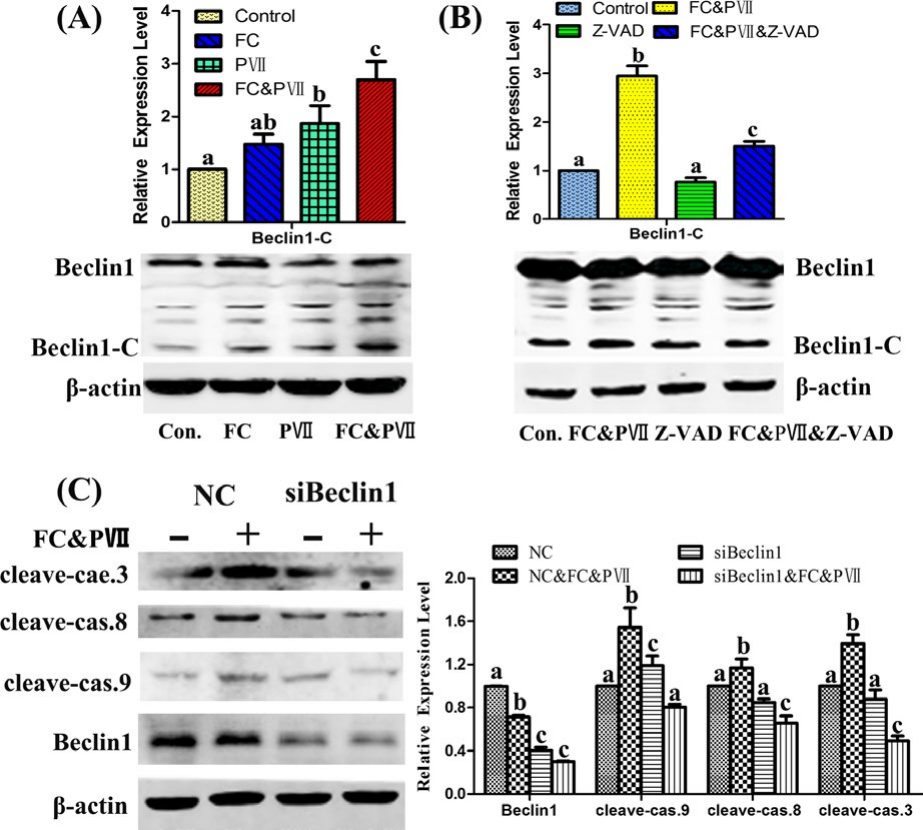
FC&PVII‐induced Beclin1 protein cleaved into Beclin1‐C. (A) Cells were treated with DMSO, FC (1 μmol/L), PVII (1 μmol/L), FC & PVII (1 μmol/L:1 μmol/L) for 24 h. Western blot analysis of autophagy proteins Beclin1 and its cleavage Beclin1‐C. (B) Cells were treated with DMSO, FC & PVII (1 μmol/L:1 μmol/L), Z‐VAD‐fmk (40 μmol/L), FC & PVII & Z‐VAD‐fmk (1 μmol/L:1 μmol/L:40 μmol/L) for 24 h. Western blot analysis of autophagy protein Beclin1 and its cleavage Beclin1‐C. (C) After transfection with a Beclin1‐targeting siRNA for 6 h, cells were exposed to FC & PVII (1 μmol/L:1 μmol/L) for an additional 24 h. Western blotting analysis of autophagy protein Beclin1 and apoptosis regulatory proteins (cleaved‐caspase 3, 8, and 9). Different letters meant significant differences between two groups (*P* < 0.05)

As the above result, combination of PVII & FC terminated autophagy and promoted apoptosis induced by FC. Previous studies showed that caspases might mediate cleavage of Beclin 1, which terminated beclin 1 inducing autophagy.^17^ As shown in Figure 6, combination of FC and PVII enhanced C‐terminal cleavage fragment of Beclin1 in NCI‐H460 cells compared with single treatment cells. Furthermore, when the cells pretreated with the pan‐caspase inhibitor Z‐VAD‐fmk, they attenuated the C‐terminal cleavage of Beclin1 induced by FC & PVII treatment (Figure 6B). As shown in Figure 6C, PVII & FC significantly increased apoptosis regulatory proteins of cleaved‐caspase 3, 8, and 9 in scramble control siRNA group. In contrast, after Beclin1‐targeting siRNA, PVII & FC reduced apoptosis regulatory proteins, which indicated that combination of PVII & FC induced apoptosis with Beclin1 dependent manner. Combination of PVII & FC induced caspases mediating cleavage of Beclin1.

## 4 DISCUSSION

Currently, drug combination has a promising and potential development prospect in treatment of various cancers. Since natural products consist of multiple components and multitargets, researchers pay more and more attention to their research and development.^4^, ^18^ Recently, after further screening different kinds of saponins from RPS in the synergistic antitumour effect on lung cancer, FC and PVII exhibited different antitumour mechanism and had a stronger synergistic antitumour activity than other combination. Hence, their synergistic antitumour mechanisms were revealed in this research.

In this study, combination of FC and PVII demonstrated a concentration‐ dependent growth inhibition in human lung cancer cells. The CI values obtained from four lung cancer cells were smaller than 1, which confirmed a synergistic interaction in the combination of FC and PVII (Figure 1, Tables 1 and 2). In order to explain the synergistic antitumour mechanism of FC & PVII, cell morphology was observed under microscopes and fluorescence microscopes. As a result, combination of FC and PVII enhanced their single proapoptotic effects, but inhibited autophagy induced by FC in NCI‐H460 cells.

To understand the proapoptosis and antiautophagy roles of PVII playing in the combination of FC and PVII, a pan‐caspase inhibitor Z‐VAD‐fmk, the autophagy initiation protein class III PI3K inhibitor 3‐MA and the later period lysosomal deacidification inhibitor CQ were used.^19^ As a result, the autophagic inhibitors could not enhance the apoptosis induced by FC (Figure 4). As we know, Bcl‐2 as a central regulator of autophagy and apoptosis functions interacted with both Beclin‐1 and Bax/Bak, respectively.^20^, ^21^ In addition, interaction of Atg5‐Atg‐12 with Fas‐ associated death domain protein can recruit and activate caspase‐8 to induce extrinsic apoptotic signaling.^22^ Autophagic inhibitors 3‐MA and CQ significantly decreased apoptosis protein expression of cleaved‐caspase‐8 and slightly increased the ratio of Bcl‐2/Bax. Therefore, inhibiting autophagy may suppress apoptosis induced by FC in NCI‐H460 cells. However, the role of PVII played in the inhibition of autophagy was not the same as that of CQ or 3‐MA.

Caspase inhibitor potentiated the effects of FC or FC&PVII on the induction of autophagy and inhibition of apoptosis in NCI‐H460 cells (Figure 5). As previously reported, PVII could trigger apoptosis via a caspase‐3‐dependent manner^14^ and induce mitochondria dysfunction.^15^ In this study, PVII also increased cleavage of caspase‐3, ‐8, and ‐9. Previous studies showed that apoptosis induced cleavage of core proteins involved in autophagy (eg, Beclin1), which inhibited autophagy. The protein fragments further triggered apoptotic cell death.^23^ Especially for caspases, they mediated cleavage of Beclin 1 to form Beclin1‐C, which terminated Beclin 1 inducing autophagy and activated apoptosis.^17^ As shown in Figure 6, combination of FC and PVII enhanced C‐terminal cleavage fragment of Beclin1 in NCI‐H460 cells compared with single treatment cells. Furthermore, when the cells are pretreated with the pan‐caspase inhibitor Z‐VAD‐fmk, it attenuated the C‐terminal cleavage fragment of Beclin1 induced by FC & PVII treatment. These results manifested that the balance between apoptosis and autophagy was associated with caspase regulation. siRNA transfection of Beclin1 experiment also indicated that PVII & FC treatment induced apoptosis with Beclin1‐dependent manner. Combination of PVII & FC induced caspases mediating cleavage of Beclin1 which further promoted the apoptosis in lung cancer cells.

In conclusion, FC and PVII have a synergistic antitumour effect through enhancing their single proapoptotic effects but inhibiting autophagy induced by FC in NCI‐H460 cells. FC and PVII activated proapoptotic elements including Bax, cleaved‐caspase‐3, ‐8, and ‐9, which triggered cleavage of Beclin1 to inhibit FC inducing autophagy and promote apoptosis. All in all, the findings would provide the foundation for the use of combination drugs in the future.

## CONFLICT OF INTEREST

We wish to confirm that there are no known conflicts of interest associated with this publication and there has been no significant financial support for this work that could have influenced its outcome.
